# First report of *Hemicriconemoides litchi* associated with *Piper sarmentosum* and revision of the genus *Hemicriconemoides* in Vietnam

**DOI:** 10.21307/jofnem-2020-074

**Published:** 2020-07-29

**Authors:** Thi Duyen Nguyen, Huu Tien Nguyen, Thi Mai Linh Le, Quang Phap Trinh

**Affiliations:** 1 Institute of Ecology and Biological Resources, Vietnam Academy of Sciences and Technology, 18 Hoang Quoc Viet, Cau Giay, 100000, Hanoi, Vietnam; 2 Graduate University of Science and Technology, Vietnam Academy of Sciences and Technology, 18 Hoang Quoc Viet, Cau Giay, 100000, Hanoi, Vietnam; 3 Nematology Research Unit, Department of Biology, Ghent University, K.L. Ledeganckstraat 35, 9000, Ghent, Belgium

**Keywords:** *H. brachyurus*, *H. cocophilus*, *H. microdoratus*, *Hemicriconemoides mangiferae*, Sheathoid nematode, Wild betel, Wild pepper

## Abstract

The taxonomic status of *Hemicriconemoides litchi*, *H. mangiferae*, and *H. strictathecatus* has been in debates for many years. In this study, a population of *H. litchi* collected from the rhizosphere of *Piper sarmentosum* is characterized using a combination of morphology and molecular data. Our results are in agreement with other authors to maintain the validity of *H. litchi* and provide sequences of *H. litchi* with correct names on GenBank. A revision on the genus *Hemicriconemoides* in Vietnam is also discussed.

The sheathoid nematodes, *Hemicriconemoides* (Chitwood and Birchfield, 1957), are root-ectoparasitic nematodes that cause damage to various crops (Sikora et al., 2018) with 54 valid species worldwide (Maria et al., 2018). In Vietnam, five *Hemicriconemoides* species have been reported including *H. litchi* (Misra and Edward, 1963), *H. mangiferae* (Siddiqi, 1961), *H. cocophilus* (Loos, 1949; Chitwood and Birchfield, 1957), *H. microdoratus* (Dasgupta et al., 1969), and *H. brachyurus* (Loos, 1949; Chitwood and Birchfield, 1957; Dasgupta et al., 1969; Nguyen and Nguyen, 2000). However, to the best of our knowledge, molecular data of *Hemicriconemoides* spp. in Vietnam are not available, and according to recent debates on the genus *Hemicriconemoides*, the evaluations for nominal species in the country are needed. Herein, a population of *H. litchi* associated with *Piper sarmentosum* Roxb. in Vietnam is characterized by morphological and molecular characterizations. Besides, a revision of the genus *Hemicriconemoides* in Vietnam is also provided.

## Material and methods

Nematodes were extracted from the rhizosphere samples of *P. sarmentosum* in Vinh Phuc province, Vietnam, using the modified Baermann tray method (Whitehead and Hemming, 1965). For morphological characterization, permanent slides were made (Nguyen et al., 2017). Photographs and measurements were obtained using a Carl Zeiss Axio Lab. A1 light microscope was used equipped with a digital camera. The 5′-end region of 28S rDNA was amplified using DP391/501 primers (5′-AGCGGAGGAAAAGAAACTAA-3′/5′-TCGGAAGGAACCAGCTACTA-3′) and analyzed using Geneious R11 (Nguyen et al., 2019). JB3/JB4 primers (5′-TTTTTTGGGCATCCTGAGGTTTAT-3′/5′-TAAAGAAAGAACATAATGAAAATG-3′) were used to amplify *COI* mtDNA gen region (Nguyen et al., 2019). References on *Hemicriconemoides* spp. in Vietnam were collected and evaluated.

## Results and discussion

### *H. litchi* Misra and Edward, 1963

#### Measurements and specimens

After Eroshenko et al. (1985) (population from Vietnam):

Details of female are as follows (*n* = 10): *L* = 480 to 520 µm; *a* = 14 to 21; *b* = 4.6 to 5.2; *c* = 13 to 15; *V* = 91 to 93%; stylet = 62 to 65 µm; *R* = 120 to 131; Rst = 20 to 22; Rex = 29 to 32; RV = 11 to 13; RVan = 5; Ran = 6 to 8.

After Nguyen et al. (2020) (population from Vietnam, in this study):

Details of female are as follows (*n* = 20): *L* = 567 ± 26 (537-609) µm, *a* = 18.3 ± 1.2 (16.8-21), *b* = 5.3 ± 0.2 (5-5.7), *c* = 28 ± 4 (24-35), *c*′ = 1.1 ± 0.1 (1-1.3), *V* = 92 ± 1 (91-93) %, VL/VB = 1.6 ± 0.1 (1.5-1.7), lip width = 11.6 ± 0.7 (10.7-12.7) µm, lip height = 5.5 ± 0.6 (4.7-6.4) µm, metenchium = 54 ± 2 (51-57) µm, telenchium = 11.4 ± 2 (9.2-14) µm, knob = 3.5 ± 0.4 (3-4.1) µm, stylet = 69 ± 3 (65-73) µm, DGO = 4 ± 1 (2.9-5.4) µm, anterior end to secretory-excretory pore = 136 ± 9 (126-151) µm, anterior end to nerve ring = 89 ± 4 (81-95) µm, pharynx = 107 ± 3 (104-113) µm, width at mid-body = 31 ± 1 (29-32) µm, width at anus = 18 ± 1 (16.5-20) µm, tail = 21 ± 3 (17-24) µm, *R* = 120 ± 6 (110-130), Rst = 18 ± 0.8 (17-19), Roes = 25 ± 1 (24-26), Rex = 32 ± 2 (30-34), RV = 10 ± 0.4 (10-11), Rvan = 4.9 ± 0.6 (4-6).

##### Remarks

**Figure 1:**
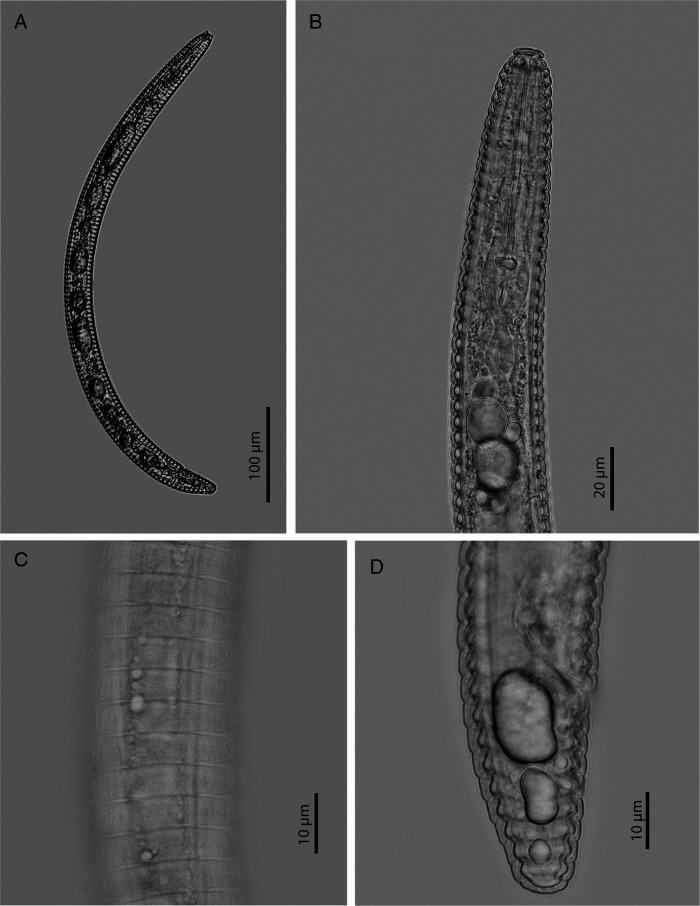
Female of *H. litchi* from Vietnam. A: Entire body; B: Pharyngeal region; C: Annuli at mid-body; D: Tail region.

**Figure 2:**
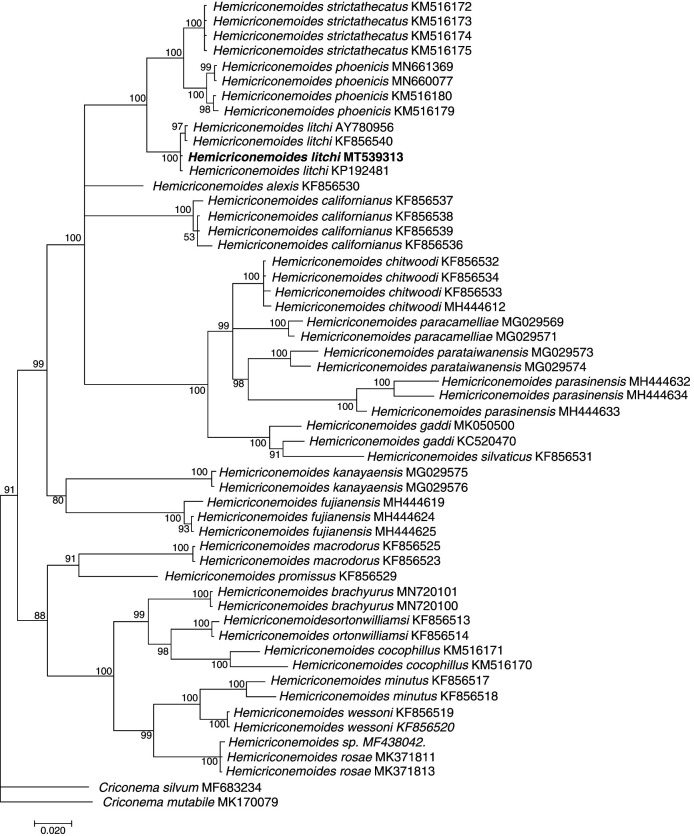
BI phylogenetic tree generated from 28S rDNA sequences (GTR + G + I model, 1 × 106 generations, 20% Burn-in). Bayesian posterior probabilities (in percentage) are given next to each node. Sequences of *H. litchi* from Vietnam are in bold font.

The female of *H. litchi* from Vietnam is characterized by having a close sheath fitting; lip region bearing two annuli with first lip annulus protruding outward; frequently curved dorsally stylet with rounded stylet knobs; secretory-excretory pore located approximately seven annuli posterior to the end of pharynx; oval spermatheca full of sperm; and tail with bluntly rounded tail tip (Fig. 1). Morphology and morphometrics of *H. litchi* from Vietnam are in agreement with the description of the type population with small variations that can be seen from other populations of *H. litchi* reported by Van Den Berg et al. (2015). For molecular characterization, the 28S rDNA sequence of *H. litchi* from Vietnam with 938 bp (accession number: MT539313) was 99.8 to 99.9% similar (1-2 bp difference) to sequences of *H. litchi* from other populations (accession number: KP192481, KF856540, AY780956). The phylogenetic tree based on 28S rDNA sequences showed that the sequence of *H. litchi* from Vietnam was grouped in the same clade (100% PP) with sequences of *H. litchi* from other populations (Fig. 2). The validation of *H. litchi* has been revised by many studies (Siddiqi, 2000; Geraert, 2010; Chen et al., 2011). Recently, a study of Van Den Berg et al. (2015) agreed with Chen et al. (2011) to maintain the validity of *H. litchi*; however, the sequences of *H. litchi* provided by the authors still appear as *H. strictathecatus* on GenBank (accession number: KF856540, KF856541, AY780956, AY780957, AY780958) confusing the identification process, especially for studies using molecular approach only. This study provides the first report of *H. lichi* associated with *P. sarmentosum* using an integrated approach of morphology and molecular tools, a 28S rDNA sequence with correct name was also submitted to GenBank. Two *COI* mtDNA sequences of *H. litchi* from Vietnam were also obtained and submitted to GenBank (accession number: MT586696, 586697). These are first *COI* mtDNA sequences of *H. litchi* on GenBank.

### *H. mangiferae* Siddiqi, 1961

#### Measurements and specimens

After Eroshenko et al. (1985) (population from Vietnam):

Details of female are as follows (*n* = 10): *L* = 450 to 560 µm; *a* = 17 to 22; *b* = 4 to 5; *c* = 17 to 24; *V* = 91 to 94%; stylet = 67 to 72 µm; *R* = 123 to 136; Rst = 18 to 20; Roes = 26 to 28; Rex = 34 to 36; RV = 10 to 13; Ran = 5 to 8.

After Germani and Anderson (1991) (population from coffee, Vietnam):

Details of female are as follows (*n* = 8): *L* = 460 ± 3 (400-500) µm; *a* = 15 ± 1.3 (13.5-16.9); *b* = 4.7 ± 0.1 (4.5-5); *c* = 20.8 ± 2.58 (16.5-28.8); *V* = 92.6 ± 0.91 (91.2-94.1) %, *V*′ = 34 ± 4.4 (28-38); *R* = 125 ± 6.6 (116-136); Rex = 34 ± 3.0 (32-40); Rv = 10 ± 1.7 (8-13); Ran = 6 ± 2.0 (4-9); Rvan = 4 ± 0.6 (3-5); VL/VB = 1.42 ± 0.14 (1.22-1.62); stylet = 63 ± 1.7 (62-65) µm.

##### Remarks

Although Chen et al. (2011) differentiated *H. mangiferae* from *H. litchi* using the combination of morphological characterizations and molecular data, Van Den Berg et al. (2015) proposed to transfer species status of *H. mangiferae* provided by Chen et al. (2011) to *H. strictathecatus*. On the contrary, Van Den Berg et al. (2015) also gave the corrections to other populations that previously was identified as *H. mangiferae*, including the revision of the population of *H. mangiferae* provided by Germani and Anderson (1991) to be conspecific with *H. litchi*. Considering the earliest conclusions of Decraemer and Geraert (1992, 1996) and, recently, Van Den Berg et al. (2015), we propose that the population of *H. mangiferae* from Vietnam provided by Eroshenko et al. (1985) should belong to *H. litchi*.

### *H. cocophilus* (Loos, 1949) Chitwood and Birchfield, 1957

#### Measurements and specimens

After Eroshenko et al. (1985) (population from Vietnam):

Details of female are as follows (*n* = 10): *L* = 460 to 500 µm; *a* = 14 to 17; *b* = 4.4 to 4.7; *c* = 13 to 14; *V* = 89 to 93%; stylet = 49 to 56 µm; *R* = 101 to 106; Rst = 11 to 12; Rex = 29 to 31; RV = 9 to 10; RVan = 2; Ran = 7 to 8.

After Nguyen (1989) (population from Vietnam, according to Nguyen and Nguyen (2000)):

Details of female are as follows: L = 0.37 mm; a = 14.6; b = 3.6; c = 11.1; V = 90.4%; stylet = 57 µm.

After Nguyen (1996) (population from Vietnam, according to Nguyen and Nguyen (2000)):

Details of female are as follows (*n* = 5): *L* = 375 to 440 (400.8) µm; *a* = 14.7 to 16.6 (15.3); *b* = 4.3 to 5.0 (4.6); *c* = 15 to 24.5 (19.9); *V* = 91.6 to 95.1 (93.1) %; stylet = 49.4 to 50.9 (50.0) µm; VL/VB = 0.8 to 1.5 (1.3); *R* = 96 to 107 (103); Rst = 14 to 15 (14.6); Roes = 23.0; Rex = 27 to 28 (27.8); RV = 8 to 11 (9.6); RVan = 2.0; Ran = 6 to 9 (7.6).

##### Remarks

Morphology of the Vietnamese populations of *H. cocophilus* is highly in accordance with the type population of *H. cocophilus*. Vietnamese population of *H. cocophilus* from Eroshenko et al. (1985) is of equal size, and other populations are of smaller size compared to the type population. These populations can be characterized by the closely fitting sheath; lip region with two annuli, first annulus smaller than second; stylet strong with anchor-shaped knobs; secretory-excretory pore situated 6 to 12 annuli posterior to end of pharynx; vulval flap 1 to 2 annuli long, distinct; anus situated about one annulus posterior to vulva; oval spermatheca with sperms; and tail with a finely rounded tip.

### H. microdoratus Dasgupta, Raski & Van Gundy, 1969

#### Remarks

Nguyen and Nguyen (2000) reported the presence of *H. microdoratus* from a forest in Gia Lai, Vietnam. The female of Vietnamese population of *H. microdoratus* is characterized by having lip region not offset from body contour, bearing two annuli, first annulus larger than second; labial disc slightly elevated, rounded; cuticular annuli 3 to 5 µm wide at mid-body; stylet knob anchor-shaped, 5 to 6 µm long; dorsal orifice gland located 3 to 5 µm posterior to stylet base; secretory-excretory pore located 97 to 105 µm from anterior end; vulva located 11 annuli anterior to tail end, vulvar sheath present; anus located 1 annulus posterior to vulva; and tail tapering to a rounded tail tip.

### *H. brachyurus* (Loos, 1949) Chitwood and Birchfield, 1957

#### Measurements and specimens

After Germani and Anderson (1991) (population from pepper, Vietnam):

Details of female are as follows (*n* = 9): *L* = 440 ± 4 (360-460) µm; *a* = 11 ± 0.6 (9.8-11.6); *b* = 4.7 ± 0.3 (4.3-5.3); *c* = 17.3 ± 4.06 (13.7-26.8); *V* = 92.4 ± 1.42 (90.8-95.5) %; *V*′ = 33.2 ± 5.2 (20-38) µm; *R* = 97 ± 5.4 (93-105); RSt = 13 ± 0.9 (12-14); ROe = 20 ± 1.4 (19-23); Rhem = 24 ± 1.5 (21-25); Rex = 29 ± 1.6 (27-31); RV = 8 ± 0.7 (7-9); Ran = 7 ± 1.0 (6-8); Rvan = 1; VL/VB = 1.18 ± 0.19 (0.71-1.33); stylet = 53 ± 3.5 (47-59) µm.

##### Remarks

All morphometrics of the Vietnamese population of *H. brachyurus* provided by Germani and Anderson (1991) are totally in agreement with the description of Loos (1949).
